# The first case of gland inclusion in an intrapulmonary lymph node: a mimic of metastasis

**DOI:** 10.1186/s12957-019-1726-1

**Published:** 2019-11-04

**Authors:** Chenglong Wang, Yijia Cao, Min Zeng, Lijuan Wang, Xiaojing Cao, Lingfeng Zou, Youde Cao

**Affiliations:** 1 Department of Pathology, Chongqing Hospital of Traditional Chinese Medicine, 6 Seventh Panxi Branch Road, Jiangbei District, Chongqing, 400021 China; 2 Department of Pathology, College of Basic Medicine, Chongqing Medical University, 1 Yixueyuan Road, Yuzhong, Chongqing, 400016 China

**Keywords:** Lung adenocarcinoma, Metastasis, Epithelial inclusion, Lymph node, Tumour staging

## Abstract

**Background:**

Lymph node inclusions are foci of ectopic tissue in lymph nodes, which were reported in different areas of the body. However, inclusions in the mediastinal lymph node are rare. Here, we report the first case of glandular inclusion within the parenchyma of the intrapulmonary lymph node in a patient with primary lung adenocarcinoma.

**Case presentation:**

A computed tomography (CT) scan showed a solid pulmonary nodule in the right upper lobe in a 44-year-old man. After a fine needle aspiration biopsy diagnosis of adenocarcinoma, lobectomy and lymph dissection were performed. Histological sections of the lung demonstrated a papillary predominant adenocarcinoma and one intrapulmonary lymph node, which displayed glandular inclusion occupying the node parenchyma. The gland inclusion was very similar to metastasis, but was formed by two layers of epithelial cells, and the abluminal cells were positive for P63, P40, and CK5/6. The patient has remained alive without recurrence and metastasis at the last follow-up before publication.

**Conclusions:**

It is very important to correctly diagnose a lymph node inclusion for proper clinical management.

## Introduction

Lymph node inclusions are foci of ectopic tissue in lymph nodes. Certainly, tumour can also develop from the ectopic tissue of lymph nodes [1]. Brooks et al. classified inclusions into three types: epithelial, naevomelanocytic, and decidual [2]. To date, a variety of different types of lymph node inclusions have been reported; however, mediastinal lymph node inclusions are rare. To our knowledge, there are only three reports about mediastinal lymph node inclusions, including one mesothelial cell inclusion [2] and two benign salivary gland tissue inclusions [3, 4]. Here, we report the first case of a glandular inclusion within the parenchyma of the intrapulmonary lymph node in a patient with primary lung adenocarcinoma.

## Case presentation

**Fig. 1:**
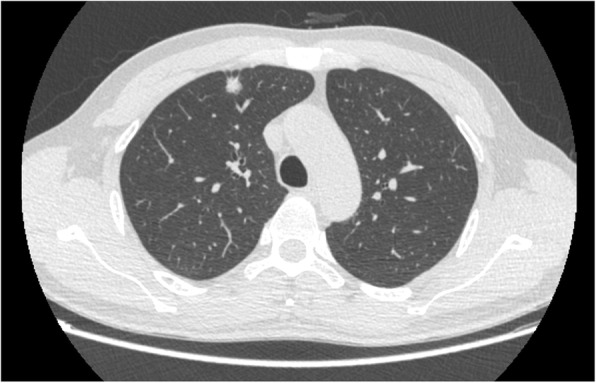
CT image showing a solid pulmonary nodule in the right upper lobe

Our patient was a 44-year-old man whose chest CT scan showed a solid pulmonary nodule in the right upper lobe a month ago (Fig. 1)*.* After anti-infective therapy, enhanced CT re-examination showed that the solid nodule was slightly larger than the previous CT scan. Due to the enlarged pulmonary nodule, the patient was admitted to our hospital on May 24, 2019. A laboratory test showed that the patient was infected with hepatitis B virus. There were no abnormal findings on other relevant examinations. The CT-guided percutaneous lung biopsy was carried out on May 29, and the biopsy was diagnosed by pathology as adenocarcinoma. The following positron-emission tomography and computed tomography (PET-CT) were performed. PET-CT images showed high uptake in the solid nodule in the right upper lobe with an SUV max value of 1.51 and pulmonary hilar lymph nodes; the latter was interpreted as a reactive increase. On June 5, lobectomy and lymph dissection were performed.

**Fig. 2:**
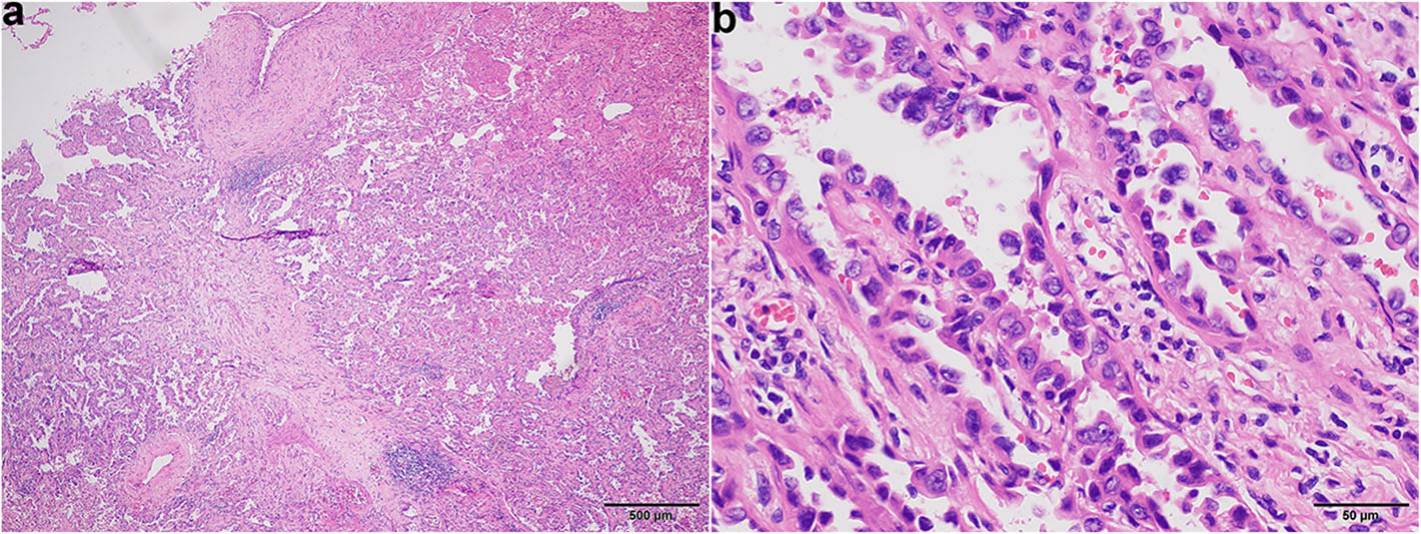
**a** The solid nodule of the right upper lobe showing a papillary predominant adenocarcinoma and the previous needle tract at the centre of the picture. **b** The tumour cells contained abundant eosinophilic cytoplasm and vacuolar nuclei with irregular contours and distinct nucleoli at high magnification

The lobectomy specimen showed a 1.2 × 0.8-cm nodule, with a tan-white, firm, and pleural indentation. Microscopic examination demonstrated an invasive adenocarcinoma with a predominant papillary pattern (Fig. 2a). The cells contained abundant eosinophilic cytoplasm and vacuolar nuclei with irregular contours and distinct nucleoli (Fig. 2b).

**Fig. 3:**
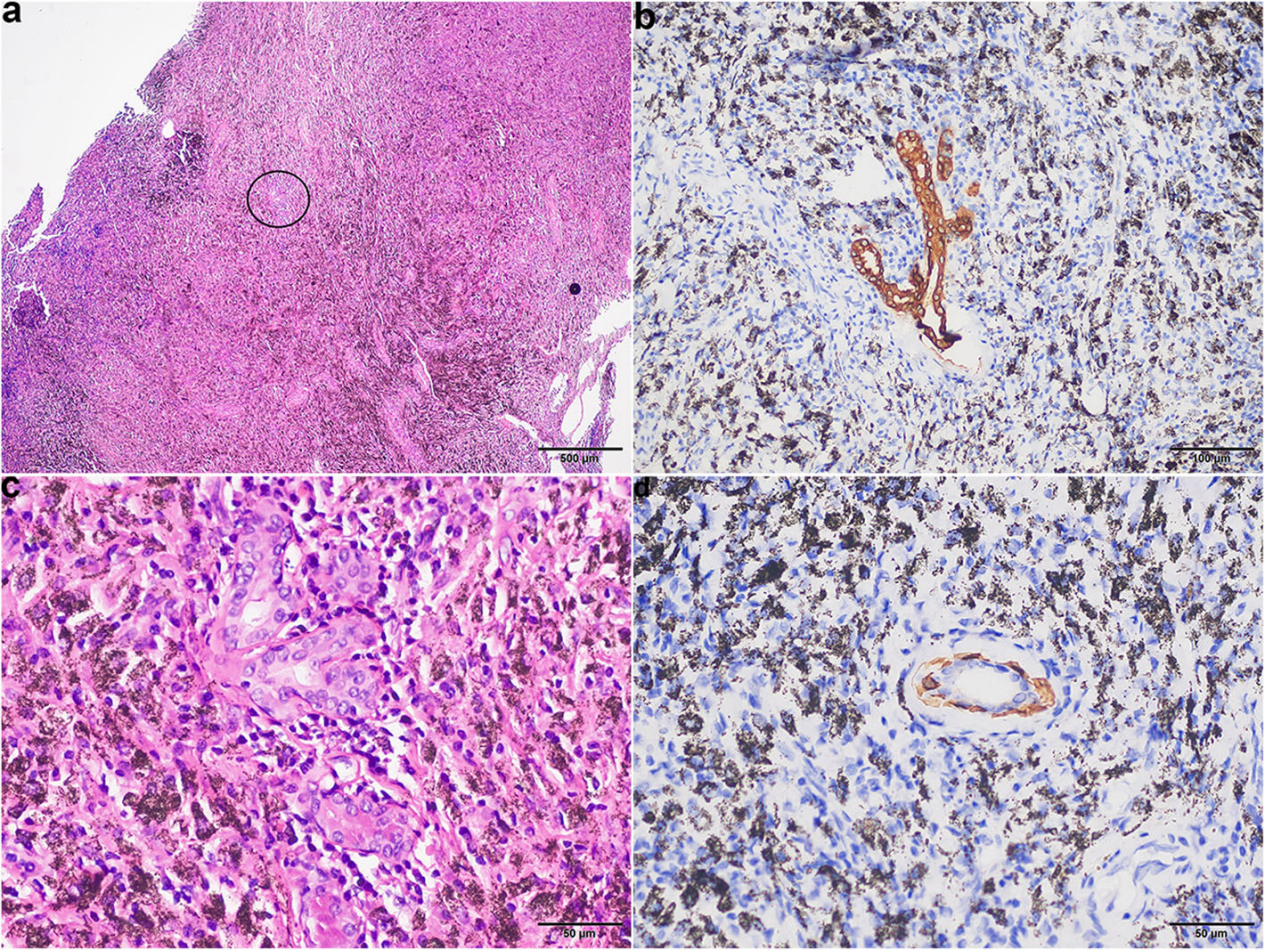
**a** A small number of glands (black circle) within the parenchyma of the intrapulmonary lymph node. **b** CK. **c** The glands contained two layers of epithelial cells, the luminal cells contained abundant eosinophilic cytoplasm and vacuolar nuclei with relatively regular contours and distinct nucleoli, and the abluminal cells showed small round hyperchromatic nuclei with indistinct cytoplasm. **d** CK5/6

In total, 28 lymph nodes were examined, ranging from 0.5 to 2.5 cm in size in the largest dimension. These lymph nodes were tan-black, without macroscopic lesions. Thirteen of the lymph nodes were designated as intrapulmonary lymph nodes. One of the intrapulmonary lymph nodes showed a few CK-positive glands within the node parenchyma (Fig. 3a, b). The glands had two layers of epithelial cells in the haematoxylin-eosin stain (HE stain) section (Fig. 3c). The luminal cells contained abundant eosinophilic cytoplasm and vacuolar nuclei with relatively regular contours and distinct nucleoli, which were very similar to pulmonary adenocarcinoma cells. The abluminal cells were small round hyperchromatic nuclei with indistinct cytoplasm and were positive for P63, P40, and CK5/6 (Fig. 3d). The patient has remained alive without recurrence and metastasis at the last follow-up before publication.

## Discusion and conclusions

In this case study, a gland inclusion in the intrapulmonary lymph node was formed by two layers of epithelial cells, and the abluminal cells were positive for P63, P40, and CK5/6. As the inclusion was not observed in the subsequent section, we could not add the myoepithelial marker to identify whether the abluminal cells were myoepithelial cells or basal cells. Many normal tissues, such as breast, prostate, tracheal mucosa, and salivary tissues, contain two layers of epithelial cells. According to embryology, the region of lymph nodes changes with different types of inclusions. For example, inclusions in cervical lymph nodes commonly are ectopic thyroid and salivary tissues, inclusions in axillary lymph nodes are ectopic breast tissue, and inclusions in pelvic lymph nodes are epithelium of paramesonephric type and decidual. The histology of inclusion in this patient was very similar to the duct part of salivary gland. As intrapulmonary lymph nodes showed gland inclusion, it is possible to think it was a duct part of the tracheobronchial submucosa gland. Engelhardt et al. showed that the pluripotent progenitor cells that exist in the human tracheobronchial surface airway epithelium have a developmental capacity for submucosa glands (SMGs) [5], and Liu et al. showed that epithelial invasion of the extracellular matrix is an important aspect of lung development and SMG morphogenesis [6]. These studies provided feasible theory for the ectopia of the tracheobronchial submucosa gland.

Generally, it is enough to identify the metastatic lesion in lymph nodes by examining the HE-stained section alone. However, in this case, it was easy to mistake the lymph node inclusion as a metastasis by examining the HE-stained section alone. The inclusion was initially diagnosed as metastasis by an experienced pathologist. The misdiagnosis might be attributed to the following reasons. First, the inclusion was formed by only a duct-like structural gland, histologically, and mild-to-moderate atypical luminal cells were very similar to pulmonary adenocarcinoma cells. In contrast to this study, the benign salivary gland tissue inclusion was easily identified in Lewis et al.’s paper [4] because the inclusion was composed of nests of salivary acini of both serous and mucinous types, rather than a salivary duct. Moreover, although many studies demonstrated that the inclusions were most commonly located within the lymph node capsule and subcapsular [4, 7, 8], the lymph node parenchyma was where the inclusion was located in this patient. Due to the atypical location, the inclusion was easily mistaken as a metastatic tumour.

With the development of radiology and the popularisation of minimally invasive surgery, an increasing number of small pulmonary nodules and suspicious lymph nodes, such as enlarged lymph nodes found during surgery and with a high uptake in PET-CT imaging, will be resected and examined pathologically. Lymph node inclusions mistaken as metastasis can lead to erroneously assigning a higher tumour stage or change the tumour character. For example, after mistaking the inclusions as metastasis, the primary lesion will be diagnosed as malignant instead of the correct diagnosis of premalignant or benign because of metastatic behaviour. It is necessary to know the potential pitfall of lymph node inclusions and avoid needlessly submitting patients to excessive and expensive treatments.

In conclusion, this paper expands the histological spectrum of benign epithelial nodal inclusions. To our knowledge, we report the first case of glandular inclusion within the parenchyma of the intrapulmonary lymph node in a patient with primary lung adenocarcinoma. The glands in intrapulmonary lymph node inclusion were formed by two layers of epithelial cells, and the abluminal cells were positive for P63, P40, and CK5/6. Although the glands are very similar to metastatic tumours, the structure of the glands in lymph nodes is different from the pulmonary adenocarcinoma. Therefore, it is undoubted that glands are benign gland inclusions in the intrapulmonary lymph node. A correct diagnosis of lymph node inclusions will benefit the evaluation of proper tumour stage and avoid needless overtreatment.

## Data Availability

Data supporting the conclusions of this study are included in this published article.

## Abbreviations

CT: Computed tomography
PET-CT: Positron-emission tomography and computed tomography
SMGs: Submucosa glands
